# Atypical right hemisphere response to slow temporal modulations in children with developmental dyslexia

**DOI:** 10.1016/j.neuroimage.2016.08.012

**Published:** 2016-12

**Authors:** Simone Cutini, Dénes Szűcs, Natasha Mead, Martina Huss, Usha Goswami

**Affiliations:** a Department of Developmental Psychology, University of Padova, Italy; b Centre for Neuroscience in Education, Department of Psychology, Downing Street, Cambridge CB2 3EB, UK

**Keywords:** fNIRs, Entrainment, Dyslexia, Phonology, Prosody

## Abstract

Phase entrainment of neuronal oscillations is thought to play a central role in encoding speech. Children with developmental dyslexia show impaired phonological processing of speech, proposed theoretically to be related to atypical phase entrainment to slower temporal modulations in speech (< 10 Hz). While studies of children with dyslexia have found atypical phase entrainment in the delta band (~ 2 Hz), some studies of adults with developmental dyslexia have shown impaired entrainment in the low gamma band (~ 35–50 Hz). Meanwhile, studies of neurotypical adults suggest asymmetric temporal sensitivity in auditory cortex, with preferential processing of slower modulations by right auditory cortex, and faster modulations processed bilaterally. Here we compared neural entrainment to slow (2 Hz) versus faster (40 Hz) amplitude-modulated noise using fNIRS to study possible hemispheric asymmetry effects in children with developmental dyslexia. We predicted atypical right hemisphere responding to 2 Hz modulations for the children with dyslexia in comparison to control children, but equivalent responding to 40 Hz modulations in both hemispheres. Analyses of HbO concentration revealed a right-lateralised region focused on the supra-marginal gyrus that was more active in children with dyslexia than in control children for 2 Hz stimulation. We discuss possible links to linguistic prosodic processing, and interpret the data with respect to a neural ‘temporal sampling’ framework for conceptualizing the phonological deficits that characterise children with developmental dyslexia across languages.

## Introduction

The speech signal carries information at multiple temporal scales, and the brain processes both slow and faster energy modulations simultaneously as part of speech encoding. Experimental studies with adults reveal that hierarchically-nested cortical oscillations at the rates of delta (~ 1–3 Hz), theta (~ 4–8 Hz), beta (~ 15–30 Hz) and gamma (> 30 Hz) track the intensity or amplitude modulations in speech in a remarkably faithful way (e.g., Luo and Poeppel, 2007, Morillon et al., 2012, Luo et al., 2010; see Giraud and Poeppel, 2012, for a recent overview). Neuronal cortical oscillations provide a mechanism for the *multi-time resolution* of the speech signal (Poeppel, 2003). Oscillating brain rhythms reflect excitability cycles, namely the concentration of neuronal electrical discharges to particular phases of a temporal cycle. This enables cell networks to align their high excitability rhythmic phase to modulation peaks in the ongoing signal, a process called phase alignment (neuronal entrainment). Phase entrainment enables the brain to encode the amplitude modulations at different temporal rates in speech *in parallel* (Doelling et al., 2014, Gross et al., 2013, Peelle et al., 2013, Park et al., 2015). Across languages, children with developmental dyslexia show impaired processing of phonology (impairments in processing speech sound structure; see Ziegler and Goswami, 2005). A neural oscillatory framework for understanding this ‘phonological deficit’ in dyslexia across languages based on atypical neuronal entrainment has been proposed: Temporal Sampling theory (Goswami, 2011).

Temporal sampling theory was motivated by the neural multi-time resolution models of speech processing developed by Poeppel and his colleagues (Poeppel, 2003, Poeppel et al., 2008). Poeppel and others (Doelling et al., 2014, Gross et al., 2013) demonstrated that acoustic events such as amplitude *rise times* (energy increases in the signal, which act as auditory “edges”) phase-reset ongoing endogenous oscillatory activity, enabling phase alignment. Children with dyslexia are impaired in rise time discrimination across languages (Goswami, 2015, for a recent summary). Accordingly, temporal sampling theory proposed that atypical neural entrainment to AMs < 10 Hz, present from birth and mediated by impaired sensory discrimination of amplitude rise times, might affect language acquisition and phonological development in dyslexia from infancy onwards. Studies of phonological development show that the emergence of ‘phonological awareness’ in all children develops in an hierarchical fashion across languages, from larger (e.g., syllables) to smaller (e.g. phonemes) units. Cortical oscillations at different temporal rates yield acoustic information relevant to this *phonological structure* of speech, with *delta* band information related to the extraction of syllable stress patterns, *theta* band information related to the extraction of syllabic information, *beta* band information related to onset-rime units (to divide a syllable into the linguistic units of *onset* and *rime*, segment at the vowel, e.g. *s-ing, st-ing, str-ing*), and *low gamma* band information related to phonetic information (see Ghitza and Greenberg, 2009, Ghitza et al., 2012, Poeppel, 2014, Leong and Goswami, 2015). There is also hemispheric specialization, with a right-hemisphere preference for slower temporal modulations (e.g., Boemio et al., 2005). As oscillatory phase entrainment is implicated in both bottom-up processing of low-level acoustic cues in the signal (Howard and Poeppel, 2010, Gross et al., 2013), and in top-down processing of high level cues such as semantic information (Peelle et al., 2013, Gross et al., 2013), children with functionally atypical phase entrainment would process the speech signal in a different way to typically-developing children. This could lead to the phonological system developing differently in children with developmental dyslexia, with lexical phonological representations encoding subtly different acoustic information.

Given that natural child-directed speech (CDS, e.g. nursery rhymes) and infant-directed speech (IDS) are highly rhythmic, accurate phase entrainment to *slower* temporal modulations might be expected to be critical developmentally for setting up the language system (Goswami, 2015). In infancy and early childhood, when semantic and pragmatic knowledge are relatively sparse, bottom-up phase entrainment to prosodic structure might be of primary importance (Kovelman et al., 2012, Goswami and Leong, 2013). The role of phase entrainment in the accuracy of speech processing by infants and children has yet to be studied in any depth. However, recent modelling of the CDS speech signal (English nursery rhymes) has revealed a core role for amplitude modulations (AMs) at the delta rate in the phonological hierarchy. Using an amplitude demodulation approach, Leong and Goswami (2015) characterised the acoustic statistical structure of English nursery rhymes as a Spectral-Amplitude Modulation Phase Hierarchy (S-AMPH) model. Their modelling revealed that the core statistical dependencies in CDS could be described by 3 nested tiers of AM which were found across all spectral bands, at temporal rates of AM centred on ~ 2 Hz, ~ 5 Hz and ~ 20 Hz (these temporal rates would correspond to neuronal oscillations in the delta, theta and beta ranges). The AM tiers were arranged hierarchically in the speech signal, with delta (2 Hz) as the master oscillator. Indeed, analyses of infant-directed speech (IDS) reveal that the greatest energy in the speech signal is in the delta band, suggestive of a core role for slow temporal modulations in initial language learning (Leong et al., 2014). The dominance of energy modulations in the delta band also provides an interesting contrast to adult-directed speech (ADS), in which theta band modulations dominate (~ 5 Hz). The modulation peak in the theta band in ADS has caused theta to be described as the master oscillator for speech processing by adults (e.g., Ghitza, 2011). Logically, however, as all studies of ADS to date have used highly literate participants, the shift in maximal energy from the delta band (in speech to infants and young children) to the theta band (in speech to adults) could reflect changes in neural speech processing that are a product of learning to read.

Available studies of oscillatory entrainment in infants and children have so far focused on non-speech stimuli, and have usually compared entrainment to amplitude-modulated noise at the two temporal rates for linguistic processing originally identified by multi-time resolution models, theta (putative syllable rate) and gamma (putative phonetic rate, see Poeppel, 2003, Poeppel et al., 2008). These non-speech stimuli typically measure neural entrainment by recording the *amplitude following response* of the brain oscillations (auditory steady state response, ASSR). Studies using electroencephalography (EEG) and functional near infrared spectroscopy (fNIRS) have revealed that even newborn (German) infants show an ASSR to amplitude-modulated noise at the contrasting rates of 3 Hz (~ delta/theta band) and 40 Hz (gamma band, Telkemeyer et al., 2009, Telkemeyer et al., 2011). Furthermore, the ASSR is right-lateralised for the slower rate in both newborn and older infants, whereas for the faster (gamma) rate the ASSR is bilateral. This suggests that asymmetric processing of auditory temporal input is a core feature of human speech processing, with preferential right-lateralized processing of slower temporal modulations present from (or before) birth. Meanwhile, a recent EEG study of Dutch-speaking 5-year-old children recorded the ASSR to speech-weighted noise stimuli amplitude-modulated at 4 Hz or 20 Hz, designated the ‘syllable’ and ‘phoneme’ rates by the authors (Vanvooren et al., 2014). Vanvooren and colleagues reported a right hemisphere preference for processing the syllable-rate modulations in these pre-reading children, and a symmetric pattern for phoneme-rate modulations.

We are only aware of one published study of phase entrainment to *speech stimuli* by children without learning difficulties. Power and colleagues designed an EEG speech paradigm based on rhythmic repetition of the syllable “ba” at a 2 Hz rate. English-speaking children either saw a ‘talking head’ repeating “ba”, so that both visual and auditory information was present (audio-visual or AV condition), saw the talking head without sound, so that only visual information was present (V), or heard the auditory stimulus stream in the absence of visual stimulation (A). The children were asked to detect occasional rhythmic violations in each condition (A, V, AV). Power et al. (2012) found significant oscillatory entrainment at the stimulation rate (delta, 2 Hz) in all three conditions, and also significant entrainment at the theta rate in the auditory and AV conditions, consistent with the predictions of multi-time resolution models of speech processing (i.e., theta entrainment was important in processing this syllabic input). Furthermore, Power et al. reported that individual differences in the strength of *theta* entrainment (measured by inter-trial coherence or phase consistency) was related to the development of reading in their typically-developing sample. Higher theta phase consistency was associated with better reading. Hemispheric differences were not measured.

Studies of oscillatory phase entrainment are more frequent in the developmental dyslexia literature, although most studies to date have utilised adult participants and have focused on the fast gamma oscillations thought to support phonetic analysis. For example, Lehongre and colleagues used amplitude-modulated white noise at rates that increased incrementally from 10 to 80 Hz and the ASSR to study neural entrainment in French-speaking adults with and without dyslexia. Of particular theoretical interest were oscillations in the low gamma band (25–35 Hz), thought to reflect optimal phonemic encoding. Both dyslexic and control participants showed significant phase entrainment, but the typical adult pattern of left-dominant gamma entrainment was shown by the control participants only. Lehongre et al. (2011) argued that their data suggested a focal (left-lateralised) impairment in the selective extraction and encoding of phonemic information in developmental dyslexia. However, it is impossible to know whether this selective impairment was present earlier in childhood, or whether it arose because of the severely reduced reading experience (and associated reduced grapheme-phoneme recoding experience) that accompanies being dyslexic (see Goswami, 2015). In a second study with French-speaking adult dyslexics using conversational speech (viewing a movie), Lehongre et al. (2013) replicated the finding of atypical left hemisphere responding to gamma band information, but did not find group differences in the neural response to theta- or delta-band information in pre-determined regions of interest (Heschl's gyrus and planum temporale). However, it is logically possible that atypical responding to slower modulations in speech may have been present earlier in development and/or in other brain regions. Meanwhile, Poelmans et al. (2012) used the same nonspeech stimuli as Vanvooren et al. (2014) to study the ASSR to 4 Hz and 20 Hz stimulation in Dutch-speaking adults with and without dyslexia. They reported a significant group x laterality effect for the 20 Hz stimulus only, also concluding that cortical processing of phoneme-rate modulations rather than syllable-rate modulations was impaired in developmental dyslexia. Again, however, whether a syllable-rate entrainment difference was present in childhood is impossible to assess.

As phoneme awareness is a *consequence* of learning to read (Ziegler and Goswami, 2005), temporal sampling theory expects phoneme-level deficits in dyslexia to be a *consequence* of impaired reading acquisition rather than a core feature of speech processing in dyslexia. Accordingly, group differences in hemispheric activity to gamma rate information would be expected to emerge during the years of schooling, when grapheme-phoneme learning (reduced in dyslexia) is being integrated into neural phonological representations. Consistent with this perspective, the sample of 5-year-olds studied by Vanvooren and colleagues included children at family risk for dyslexia, yet Vanvooren et al. (2014) found no differences regarding entrainment to the 20 Hz stimulus between these “at risk” pre-reading children and the other pre-reading children in the sample. Nevertheless, note that the transparency of the orthography that is being learned may also affect the balance of hemispheric processing of temporal modulations at different rates. Orthographic consistency has measurable developmental effects on oral speech processing, particularly at the phonemic level (e.g., Goswami et al., 2005). Accordingly, it is likely that neural speech processing also changes in response to learning to read, and that it changes in subtly different ways in response to learning different orthographies.

Phase entrainment at the theoretically-important delta rate in dyslexia has been studied using nonspeech stimuli in both adults and children. Hämäläinen and colleagues played amplitude-modulated white noise at 4 temporal rates (2 Hz, 4 Hz, 10 Hz, 20 Hz) to English-speaking adults with and without dyslexia in an unattended listening paradigm. On the basis of temporal sampling theory, they predicted group differences in oscillatory phase entrainment at the slower AM rates (2 Hz, 4 Hz) in the right hemisphere. The data indeed showed significantly reduced phase entrainment by the dyslexic participants in right hemisphere auditory networks, but for the 2 Hz rate only (Hämäläinen et al., 2012). There was also significantly weaker right hemisphere entrainment overall (adding across modulation rates) for the dyslexics, and significantly stronger entrainment to the 10 Hz rate in the *left* hemisphere, a finding which was not predicted. This could indicate compensatory entrainment at faster temporal rates in dyslexia, a finding also reported (bilaterally) by Lehongre et al. (2011) for rates > 50 Hz. In a second study with English-speaking adults using EEG and an attended button-press paradigm, Soltész and colleagues compared phase entrainment to a rhythmic tone stream delivered at 2 Hz in dyslexic and control participants (Soltész et al., 2013). Soltész et al. found that entrainment was significantly reduced in the dyslexic participants, even though they were as fast and as accurate as the control adults in the button-press paradigm. Whereas the control participants showed faster responses in the rising phase of the oscillation, as expected, the dyslexic participants showed no relationship between oscillatory phase and behaviour. Most recently, a study in Spanish has compared the ASSR to AM noise delivered at delta, theta and gamma rates in *both* children and adults with dyslexia (Lizarazu et al., 2015). Group differences in the phase locking values for both adults and children with dyslexia in comparison to control participants were found, at both theta (4 Hz) and gamma (30 Hz) rates. Greater right-lateralized responding to 4 Hz AMs was significantly related to reading rate for the control participants only (reading accuracy was already at ceiling in this transparent orthography, even for the dyslexic children). No group differences were found at the delta rate for these non-speech stimuli in this syllable-timed language, however.

Finally, two studies of neural entrainment by children with dyslexia are available that have used speech stimuli. Power et al. (2013) administered the AV rhythmic speech paradigm developed by Power et al. (2012) to English-speaking children with dyslexia. They reported that, compared to age-matched control children, the children with dyslexia showed a *different preferred phase* of entrainment in the delta band, in response to both the auditory and the auditory-visual stimulus streams. A different preferred phase of entrainment implies enhanced neuronal phase alignment in dyslexia at *less informative* temporal points in the speech signal. For example, if the high excitability phase of the delta oscillation does not align temporally with the modulation peaks in the AMs in the speech signal, then theoretically this would affect children's ability to extract linguistic structure, particularly regarding prosodic information and syllable stress patterns. Impaired representation of speech envelope information in the delta band would also have cascading effects for other linguistic levels (syllable, onset-rime, phoneme) via the AM oscillatory hierarchy in the speech signal (see Leong and Goswami, 2015).

In Spanish, Molinaro et al. (in press) reported an MEG study of sentence processing by adults and children with dyslexia. Many of the participants were the same individuals studied by Lizarazu et al. (2015), who did not find a delta entrainment difference by group to AM noise. Molinaro et al. found that both the adults and children with dyslexia showed impaired oscillatory entrainment to speech in the delta band, with reduced delta synchronisation originating in right primary auditory cortex (Molinaro et al., 2016). Overall, these studies suggest that atypical entrainment *to speech* is present in the delta band in children with dyslexia who are learning to read both English (a stress-timed language) and Spanish (a syllable-timed language), and that it does not ameliorate over development in either language.

In the current study, we set out to measure whether English-speaking children with developmental dyslexia would show atypical right hemisphere entrainment for slower temporal modulations, while showing equivalent entrainment to control children for faster temporal modulations, the first such investigation in English. We followed Telkemeyer et al. (2009) and utilised fNIRS. Like functional magnetic resonance imaging (fMRI), fNIRS monitors hemodynamic changes in the cerebral cortex (Cutini et al., 2012, for a review). However, whereas the blood-oxygen-level-dependent (BOLD) signal of fMRI is gathered from the paramagnetic properties of deoxyhemoglobin (HbR), fNIRS is based on the intrinsic optical absorption of blood. As a result, fNIRS can simultaneously record the variations of HbR and oxygenated hemoglobin (HbO) concentrations, with a much higher temporal resolution, thereby potentially providing a richer picture of cortical hemodynamics compared with fMRI (see, e.g., Cutini et al., 2014a, Cutini et al., 2014b). fNIRS is also tolerant of motion, making it particularly suitable for studying children. We collected hemodynamic data in a passive listening paradigm based on AM noise presented at two temporal rates, 2 Hz and 40 Hz. We predicted that the children would show a bilateral cortical response to the 40 Hz stimulus, but a right-lateralized response to the 2 Hz stimulus. We also expected that children with dyslexia would show typical neural responding in the 40 Hz condition, coupled with atypical neural responding in the 2 Hz condition.

## Methods

### Participants

36 children took part in the study, of whom 18 had a statement of developmental dyslexia from their local education authority and/or showed severe literacy and phonological deficits according to our own test battery. The participants were drawn from a larger cohort of children participating in a longitudinal study of auditory processing in developmental dyslexia (see Goswami et al., 2013), and comprised all children of similar age and IQ who gave informed consent for fNIRS. Data from 3 participants in each group were discarded because they were too noisy for HbO analysis, leaving 15 children in each group, whose data form the basis of the current report. The 15 children with dyslexia (6 male) had a mean age of 12 years 11 months, a mean standardised full scale IQ (WISC short form) of 108.3 (standardised mean of this test = 100), and no diagnoses of any additional learning difficulties (e.g. ADHD, dyspraxia, SLI). The 15 chronological age (CA) matched controls (7 males) had a mean age 12 years 10 months and a mean FSIQ of 106.0. Further details of children's performance on the standardised and other tests used is given in Table 1. All of the children had English as a first language and before participating, received a short hearing screen using an audiometer. Sounds were presented in both the left or right ear at a range of frequencies (250, 500, 1000, 2000, 4000, 8000 Hz), and all participants were sensitive to sounds within the 20 dB HL range. Parental informed written consent was obtained for all participants. The study received ethical approval from the Cambridge Psychology Research Ethics Committee.

### Standardised tests of reading, mathematics, vocabulary and IQ

Children received the single word reading and mathematics subscales of the British Ability Scales (BAS, Elliott et al., 1996), the British Vocabulary Scales (Dunn et al., 1982), and the short form of the Wechsler Intelligence Scales for Children (WISC III, comprising the picture arrangement, block design, similarities and vocabulary subscales; Wechsler, 1992). These four subscales of the WISC yield an estimate of full-scale IQ (pro-rated, see Sattler, 1982). As full-scale IQ was assessed at the beginning of this longitudinal project, 4 years before the current test point, we also assessed current non-verbal IQ by administering the picture arrangement WISC subscale. The children also received two sub-scales (Inattention, Hyperactivity), of the standardised Barkley scale of attention (Barkley & Barkley and Murphy, 1998). As shown in Table 1, the children were matched for IQ, receptive vocabulary, mathematics attainment and for the measures of attention and hyperactivity, but were not matched for reading attainment. The children with dyslexia were on average showing reading levels 34 months behind the typically-developing CA controls.

### Experimental phonological and rise time psychoacoustic tasks

Three experimental measures of phonological processing were administered as part of ongoing testing in the year that fNIRS was recorded, as were three psychoacoustic measures of sensitivity to non-speech amplitude envelope rise time. Group performance in each case is shown in Table 1. The tasks comprised:

#### Phoneme deletion

In this task, digitized speech created from a native female speaker of standard Southern British English was used to present 18 pseudowords (including 3 practice words), followed in each case by a target phoneme contained in the pseudoword. Participants were asked to produce the pseudoword omitting the target phoneme (e.g. *Say “bice” without the “b”; Say “splow” without the “p”*). Phonemes were deleted from a variety of positions within the pseudoword (initial, medial, final). This was an abbreviated version of a similar deletion task designed by McDougall et al. (1994), as used by Pasquini et al. (2007). Scores out of 15 were used in the analyses.

#### Phonological short-term memory (PSTM)

The children heard 4 monosyllabic consonant-vowel-consonant words presented by computer through headphones using digitized recordings of speech produced by a female native speaker of Standard Southern British English (e.g., *type, rib, nook, bud*; task originally used by Thomson et al., 2005). The children were required to repeat back the words as spoken. Sixteen trials were presented in total, 8 comprising items drawn from dense phonological neighbourhoods, and 8 trials comprising items drawn from sparse phonological neighbourhoods. The total number of items reported correctly out of 64 was used in the analyses.

#### Rapid Automatized Naming (RAN)

In the RAN task, children were asked to name line drawings of two sets of familiar objects (first set: *cat, shell, knob, zip, thumb*; second set: *web, fish, book, dog, cup*; see Richardson et al., 2004). For each set, children were first introduced to the names of the pictures and then shown a page with the same pictures repeated 40 times in random order. The children were asked to produce the names as quickly as possible. Average naming speed across the two lists in seconds was used in the analyses.

#### Amplitude rise time (1 Rise AXB)

This was a psychoacoustic amplitude rise time discrimination task presented in AXB format. Three 800 msec tones were presented on each trial, with 500 msec ISIs. Two (standard) tones had a 15 msec linear rise time envelope, 735 msec steady state, and a 50 msec linear fall time. The third tone varied the linear onset rise time, with the longest rise time being 300 msec. Children were introduced to three cartoon dinosaurs. It was explained that each dinosaur would make a sound and that the child's task was to decide which dinosaur's sound was different from the other two and had a softer rising sound (longer rise time). The child then participated in five practice trials. Feedback was given after every trial by the computer software. During the practice period this was accompanied by further verbal explanation and reinforcement by the researcher. Up to 40 experimental trials were then administered.

The psychoacoustic stimuli in this and the following two rise time tasks described below were presented binaurally through headphones at 75 dB SPL. Earphone sensitivity was calculated using a Zwislocki coupler in one ear of a KEMAR manikin (Burkhard and Sachs, 1975). The dinosaur programme used an adaptive staircase procedure (Levitt, 1971) with a combined 2-up 1-down and 3-up 1-down procedure; after 2 reversals, the 2-up 1-down staircase procedure changed into 3-up 1-down. The step size halved after the 4th and 6th reversal. A test run typically terminated after 8 response reversals or alternatively after the maximum possible 40 trials. The threshold score achieved was calculated using the mean of the last four reversals.

#### Rise duration rove

This was exactly as the 1 Rise AXB task, except that the duration of each stimulus varied randomly across the experiment. This was done by randomly roving the duration of the steady state portion of the stimulus from 450 msec to 735 msec. If an amplitude envelope is always 800 msec long with a 50 msec fall time (as in the 1 Rise AXB task), and the rise time is either 15 msec or 300 msec, then the steady state portion of the first stimulus will be 735 msec whereas for the second it will be 450 msec. It is thus possible that children could discriminate between the rise time stimuli on the basis of the difference in steady state duration. By roving duration, we eliminated this alternative cue.

#### 1 Rise 2IFC

This used the same stimuli as the 1 Rise AXB task above, but presented them in a 2IFC format. Two 800 msec tones were presented in a random order in each trial, with 500 msec ISI. One (standard) tone had a 15 msec linear rise time envelope, 735 msec steady state, and a 50 msec linear fall time. The second tone varied the linear onset rise time, with the longest rise time being 300 msec. Children were introduced to two cartoon dinosaurs. It was explained that each dinosaur would make a sound and that the child's task was to decide whether the sounds were different

### AM Stimuli for fNIRS

The stimuli comprised 4 blocks of amplitude-modulated white noise, as used by Hämäläinen et al. (2012). There were 2 blocks of 5 min stimulation at each AM rate used (2 Hz and 40 Hz), presented sequentially in a semi-random order. During each 5 min block, 15 s periods of stimulation were interspersed with 15 s periods of silence. The children were watching a silent video during data acquisition.

### fNIRS instrumentation

The recording optical unit was a multi-channel continuous wave fNIRS instrument (ETG-4000 Hitachi Medical Corporation, Tokyo, Japan). The present investigation made use of 32 light sources (semiconductor laser diodes: 16 emitting light at 695 nm, and 16 at 830 nm) and 14 photo-detectors (Avalanche photodiodes). The sources emitted light onto the participant's scalp. The light reflected out of the scalp after scattering through the brain was captured by the highly sensitive photo-detectors. Afterwards, the optical signals recorded by the detectors came through a switching circuit set according to the probes' configuration, and were separated by a lock-in amplifier locked to the modulation frequency of the light source. Concentration changes (Δ) in HbO and HbR (mmol × mm) were calculated based on a modified Beer–Lambert approach (Cope and Delpy, 1988), that enables estimation of the relative concentration as a function of total photon path length and light intensity.

#### Probe arrangement

**Fig. 1:**
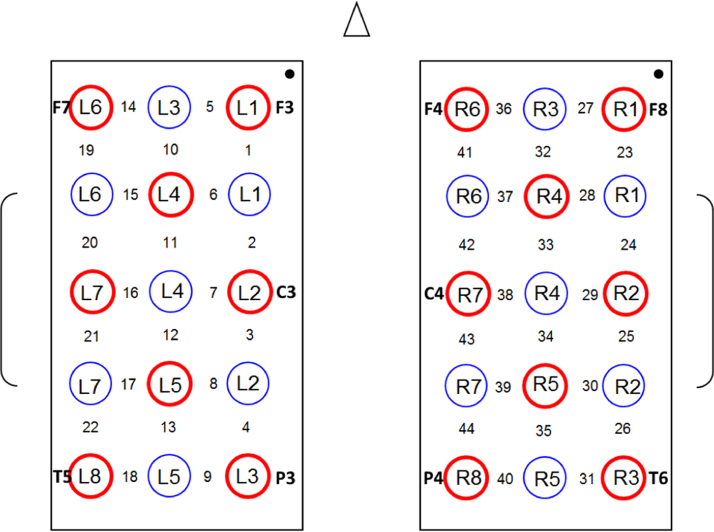
A schematic illustration of the probe placement. Red circles represent sources and blue circles represent detectors. Numbers between the sources indicate the channel, with the probe arrangement projected onto an EEG 10–20 configuration, with EEG channels noted in bold.

A single-distance probe arrangement (e.g., Cutini et al., 2008, Cutini et al., 2014a, Cutini et al., 2014b) was adopted: the space between each source/detector pair (hereafter, channel) was L = 30 mm, in order to equate the channels for optical penetration depth into the cortical tissue (Franceschini et al., 2000); the probe configuration provided 44 channels (see Fig. 1). Each source location comprised two source optical fibers, one for each wavelength. Sources and detectors were held in place on the child's head using two plastic arrays of optode holders in a rectangular shape. The position of the optodes closely resembled one of the 3 × 5 virtual registrations (e.g., Tsuzuki et al., 2007) provided by the Jichi Lab (http://www.jichi.ac.jp/brainlab/virtual_registration/Result3x5_E.html): the F7_Ch05_Hor_3x5 for the left hemisphere (see http://www.jichi.ac.jp/brainlab/virtual_registration/images/others3x5/F7_Ch10_Hor_3x5.png), and the F8_Ch20_Hor_3x5 for the right hemisphere (see http://www.jichi.ac.jp/brainlab/virtual_registration/images/others3x5/F8_Ch20_Hor_3x5.png). Virtual registration is based on simulations in place of physical measurements for optode positioning and allows the normalization of the registered positions in stereotaxic space. As a result, this procedure provides the most likely Montreal Neurological Institute (MNI) coordinate values of the optodes (the MNI represents the most popular coordinates system currently used in fMRI). This probe arrangement enabled us to record hemodynamic activity bilaterally from parietal and temporal brain regions. The cortical regions underlying each optode and channel were estimated using the LONI Probabilistic Brain Atlas (LPBA40, Shattuck et al., 2008).

The holder placement was performed by using a specific set of 10–20 positions (Jasper, 1958) as reference points (10–20 points are cranial landmarks typically used in electroencephalography). For the left hemisphere, sources L1, L2, L3, L6 and L8 were placed as close as possible to F3, C3, P3, F7 and T5, respectively. For the right hemisphere, sources R6, R7, R8, R1 and R3 were placed as close as possible to F4, C4, P4, F8 and T6, respectively. This procedure allowed us to place the holder in a consistent way across participants. Note that the precision achieved by virtual registration procedures yields a worst-case average error within the spatial resolution of the fNIRS instrument, and its consistency is supported by different methodological studies (Okamoto et al., 2004, Okamoto and Dan, 2005, Tsuzuki et al., 2007, Cutini et al., 2011).

### fNIRS signal processing

The following series of operations were performed in the analyses. The data were filtered with a 3rd order Butterworth band-pass filter, which is commonly used in fNIRS data processing (e.g., Cooper et al., 2012, Brigadoi et al., 2014) to reduce low-frequency oscillations and cardiac oscillations (0.01–1 Hz). Individual hemodynamic responses were segmented into 31 s trials starting from 1 s before the stimulus onset to 30 s after, and each trial was zero-mean corrected by subtracting the mean intensity of the optical signal recorded during the 31 s period. For each trial the standard deviation (SD), the maximum value and the minimum value were calculated: trials with SD > 0.5, maximum value > 1.5 or minimum value >− 1 mmol × mm were classified as contaminated by artifacts and then rejected (< 5%). Channels with excessively noisy data (i.e., those channels containing more than half of discarded trials) were excluded from further analysis (< 5%). Subsequently, trials of the same condition were averaged; the averaged hemodynamic response was smoothed with a Savitzky-Golay filter (Savitzky and Golay, 1964), with polynomial order equal to 3 and frame size equal to 3 s and was baseline-corrected by subtracting from the overall hemodynamic response the mean intensity of the signal in the time interval between the onset and 1 s before. Mean HbO and HbR concentrations indices (i.e., mean value between 5 and 25 s interval after trial onset) were extrapolated from the resulting hemodynamic response profiles. Repeating these operations for each child and condition allowed us to generate distinct optical maps for the two groups. For the sake of completeness, the optical maps for 2 Hz and 40 Hz against baseline were computed for both groups: all the maps showed a broad bilateral activity in most of the channels.

## Results

As will be recalled, a priori we expected to find a group difference in hemodynamic response for the 2 Hz modulation rate but not for the 40 Hz modulation rate.

To explore the impact of modulation rate on the two groups, we compared the *differential* pattern of activity for the two modulation rates between the CA controls and the children with dyslexia. We calculated the *difference* in HbO and HbR between the two modulation rates for each channel and each participant, and then performed a channel-wise series of unpaired *t*-tests aimed at comparing the differential activity in the group of children with dyslexia to that of the control group. Such comparison is formally described for both HbO and HbR as:

(HbO dyslexic 2 Hz − HbO dyslexic 40 Hz) vs. (HbO controls 2 Hz − HbO controls 40 Hz).

(HbR dyslexic 2 Hz − HbR dyslexic 40 Hz) vs. (HbR controls 2 Hz − HbR controls 40 Hz).

All the channel-wise analyses were corrected for multiple comparisons using a false discovery rate (FDR, Singh and Dan, 2006) with *q* = 0.1. The statistical values that survived multiple comparison correction were used to create optical maps utilising the following series of operations. The statistical score of each channel was mapped onto an overlay map (1 mm^3^ voxel size) at the correspondent midpoint expressed in Montreal Neurological Institute(MNI) coordinates, using the NIfTI toolbox (Neuroimaging Informatics Technology Initiative, nifti.nimh.nih.gov/). A Gaussian blurring filter (SD = 10 mm) was then applied to the overlay map to approximate the area covered by each channel. Finally, the resulting map was overlaid onto the reference brain using the MRIcron software (http://www.mccauslandcenter.sc.edu/mricro/mricron/).

**Fig. 2:**
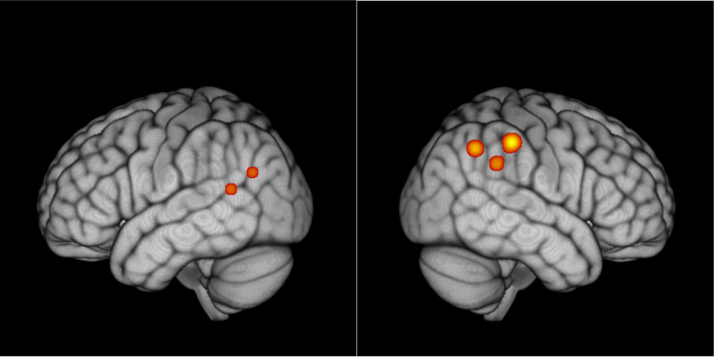
Statistical maps obtained by the series of t-tests aimed at detecting differential activity between the two groups at the modulation rates used for stimulation. The figure on the left depicts the left hemisphere, the figure on the right depicts the right hemisphere.

The series of unpaired *t*-tests for HbO concentration revealed an asymmetric bilateral pattern of regions that were more active in the children with dyslexia than in the CA controls for 2 Hz with respect to 40 Hz (see Fig. 2). Significant differences were found for the left superior temporal gyrus and the left angular gyrus (channels 9 and 13); while in the right hemisphere, the difference was almost entirely confined to the right supramarginal gyrus and right angular gyrus (channels 39, 43, 44). The involvement of left hemisphere language areas in this differential activity between the two groups suggests that our non-speech stimuli were indeed activating the neural speech processing system. It also suggests a different balance of processing for slower versus faster temporal modulations by hemisphere for the two groups, which is driven by both hemispheres.

**Fig. 3:**
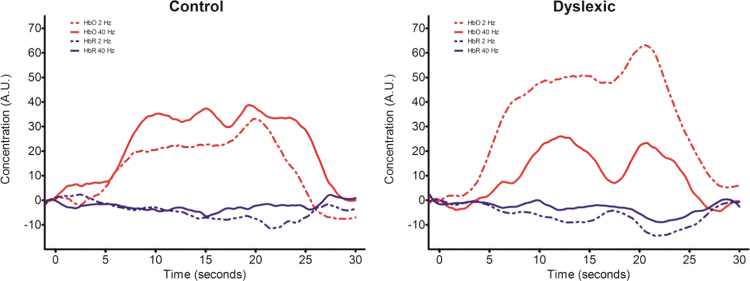
Time courses for HbO (red) and HbR (blue) concentrations for the control group (left) and the dyslexic group (right). Solid lines refer to 40 Hz, while dashed lines refer to 2 Hz. The hemodynamic response of the dyslexic group to 2 Hz stimulation is clearly larger than that of the control group.

An example of the hemodynamic response profile across the different groups and modulation rates is shown in Fig. 3, observed in the right parietal lobe (channel 43), the channel with the highest *t* statistic. It can be seen that the HbO response to the 2 Hz modulation rate was larger relative to the response to the 40 Hz modulation rate for the children with dyslexia, while the visual inspection denoted the opposite pattern (although not corroborated by statistical significance) for control children. Although the response profile of HbR was broadly consistent with HbO, no significant group differences emerged for HbR, probably because of the low signal-to-noise ratio. No further analysis for each modulation rate separately was thus performed for the HbR concentration.

To explore further the finding that the HbO response to the 2 Hz modulation rate was larger than the response to the 40 Hz modulation rate only for the children with dyslexia (see Fig. 3), we then performed an analysis for the HbO concentration on a single channel basis, aimed at identifying the core region that differed between the children with dyslexia and the control group. For each channel, we performed a mixed ANOVA with modulation rate as the within-participant factor (2 levels: 2 Hz and 40 Hz) and group as the between-participant factor (2 levels: control and dyslexic). We then collated those channels exhibiting a frequency x group interaction, which signified a differential effect of modulation rate between the two groups.

**Fig. 4:**
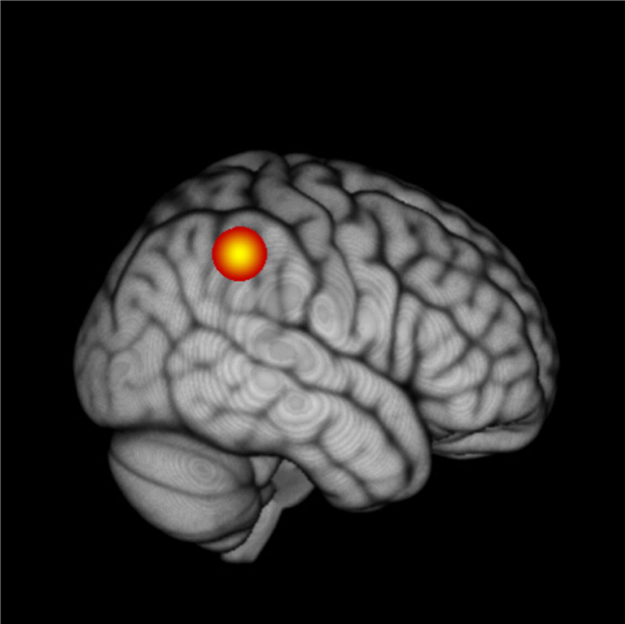
The statistical map showing the interaction between group and frequency based on the pooled activity of channels 43 and 44.

Following these procedures, only the HbO response of channels 43 and 44, two adjacent channels in the right parietal lobe, revealed a significant interaction after multiple comparison correction (ch. 43: *F*(1,28) = 16.64, *p* < 0.005; ch. 44: *F*(1,28) = 11.11, *p* < 0.005). Given the proximity of the two channels, we collapsed their activity to increase the robustness of the signal. Fig. 4 depicts the *F* value (16.169) of the interaction (group x frequency) of the pooled activity between channels 43 and 44. According to LPBA40, the region underlying the middle point between the two channels was the supramarginal gyrus. The results of a t-test between the pooled activity confirmed that dyslexic and control groups were significantly different for the 2 Hz stimulation, *t*(28) = 1.95, *p* < 0.05 (one-tailed). There were no group differences for the 40 Hz stimulation. The significant interaction refines the previous results, suggesting that this different balance of processing slower versus faster temporal modulations by group is driven primarily by the right supramarginal gyrus (Fig. 3).

Accordingly, the pooled hemodynamic HbO response values for channels 43 plus 44 at the 2 Hz and 40 Hz modulation rates respectively for each child were used for a set of correlational analyses with the behavioural measures. On the basis of temporal sampling theory, individual differences in the hemodynamic response to 2 Hz stimulation would be expected to be related to individual differences in children's sensitivity to amplitude envelope rise time, and to their language and reading development. Accordingly, we ran a partial correlation analysis utilising these behavioural measures, controlling for age and for non-verbal IQ in the year that fNIRS data were collected. The results are shown as Table 2.

Inspection of the table shows that the hemodynamic response at 2 Hz was significantly associated with basic sensory processing of amplitude rise time and with vocabulary and reading development. Greater concentrations were related to higher (= poorer) sensory thresholds and to poorer reading and vocabulary levels respectively, as would be expected. The hemodynamic response at 2 Hz was not related to individual differences in academic performance in general (standardised mathematics performance) nor to individual differences in attention. Associations with the phonological variables failed to reach significance. The hemodynamic response at 40 Hz did not show any significant relationships with the language, reading or rise time measures. This is consistent with the theoretical proposals of TS theory, that phase entrainment to faster temporal modulations (40 Hz) is not causally related to the impairments in reading and language development that characterise children with dyslexia.

## Discussion

The present study investigated neural phase entrainment to AM-noise delivered at two temporal rates, 2 Hz and 40 Hz. The participants were English children aged on average 12 years, half of whom had a specific reading difficulty (developmental dyslexia) but no other academic impairments. On the basis of the prior literature, we expected to observe a bilateral hemodynamic response to the 40 Hz stimulation, and a right-lateralised response to the 2 Hz stimulation. On the basis of our prior data (Power et al., 2013) and temporal sampling theory (Goswami, 2011, Goswami, 2015), we expected a right-lateralised atypical hemodynamic response for the children with dyslexia to the 2 Hz modulation rate only.

Consistent with these predictions, we found a significant difference between the dyslexic and the control children in the hemodynamic response to the 2 Hz stimulation, which was focused in the right supramarginal gyrus (Fig. 3). This region has long been associated with linguistic function, for example via the phonological-articulatory loop (Rauschecker and Scott, 2009). The location of the group difference found here suggests atypical function of the dorsal stream of speech processing in dyslexia, thought to be integral to speech perception and to auditory-motor speech development (Hickok and Poeppel, 2007). For example, the right supramarginal gyrus is implicated in the explicit processing of speech rhythm by adults (Geiser et al., 2008). Speech rhythm processing is known to be impaired in children with dyslexia (Goswami et al., 2013). The right hemisphere action-perception network was also found to be involved in the processing of speech prosody in a study combining fMRI and TMS (Sammler et al., 2015). Sammler et al. proposed a key role for an area bordering BA 44 and 45 in the explicit labelling of prosodic contours. Explicit prosodic awareness is also impaired in children with dyslexia (Goswami et al., 2010). Regarding developmental studies, children born prematurely, who are at known risk for language deficits, show increased connectivity as teenagers between right hemisphere dorsal language areas and Wernicke's region, with stronger connectivity linked to poorer developmental outcomes (Myers et al., 2010). Meanwhile, dyslexic teenagers show significantly reduced gray matter in the right supramarginal gyrus compared to typically-developing controls (Kronbichler et al., 2008). The conservative interpretation of our data is that the right hemisphere location identified here is related to the efficiency of processing the slower temporal modulations in speech and to the extraction of perceptual information relevant linguistically to speech rhythm and prosody. Altered hemispheric asymmetry in children with dyslexia has also been reported in an MEG study presenting diphones (ba-da) for passive listening (Heim et al., 2003). In that study, the source locations of the P100m differed between children with dyslexia and control children, with the dyslexic children showing deviant right hemisphere organization.

The hemodynamic response to 40 Hz stimulation did not show any group differences, which is also consistent with temporal sampling theory (Goswami, 2011). In the temporal sampling framework, it is proposed that processing of faster temporal modulations is not impaired in children with dyslexia *prior to learning to read* (Goswami, 2011). Rather, phonetic rate processing is *atypical* in children with dyslexia (e.g., possibly more precise), because like young infants, children with dyslexia may continue to perceive allophones, which requires fine-grained temporal sensitivity (phonemic over-sampling, see also Giraud and Poeppel, 2012). Accordingly, children with dyslexia may retain non-native speech contrasts, complicating the process of reading acquisition in alphabetic languages (Serniclaes et al., 2004). In the current study, analysis of the *differential* activation for the two modulation rates by group (Fig. 4) revealed an asymmetric bilateral pattern of regions that were more active in the children with dyslexia than in the control children. Significant group differences were found for relative activation in two areas involved in linguistic processing: the left temporal gyrus (channels 9 and 13) and the right supramarginal and angular gyri (channels 39, 43, 44). The temporal gyrus is part of the larger speech processing system located in the superior temporal sulcus and the inferior parietal lobule, sometimes described as ‘Wernicke's area’. Wernicke (1874) designated superior temporal gyrus and the inferior parietal lobule as the neural locus of speech perception. The involvement of these areas in our task when differential activation to both modulation rates was considered by group suggests that the task was indeed activating the neural speech processing system.

When we explored significant relations between the hemodynamic response to the two modulation rates and children's language and reading outcomes, a series of significant associations were found for the 2 Hz modulation rate only. Individual differences in the hemodynamic response to 2 Hz were significantly related to all the measures of amplitude rise time sensitivity that we administered, to the development of receptive vocabulary, and to reading development. Individual differences in response to the 2 Hz modulation rate were not related to mathematical performance nor to attention. These findings are consistent with the temporal sampling framework for developmental dyslexia (Goswami, 2011). The data are also consistent with multi-time resolution models of speech processing (Hickok and Poeppel, 2007, Poeppel, 2003, Ghitza and Greenberg, 2009), which predict that slower modulations should preferentially activate right-hemisphere neural networks. Our data are suggestive of functionally-atypical right-lateralised processing of slower temporal modulations in children with developmental dyslexia. Although the stimuli in the current study were non-speech, we were able to measure neural encoding of speech in the same sample of children in a later study, using semantically unpredictable noise-vocoded sentences (Power et al., 2016). Power et al. (2016) utilised a reverse engineering approach, in which the speech envelopes of the sentences were estimated from the neural (EEG) response (via envelope reconstruction, a form of speech resynthesis, see O'Sullivan et al., 2015). This enabled a direct measure of the quality of children's phonological representations. Power et al. found that the children with dyslexia had significantly poorer speech encoding in the 0–2 Hz band compared to both age-matched control participants (again, a subset of those children tested here) and also in comparison to *younger reading-level matched* (RL) control children. Inclusion of an RL-matched control group helps to determine whether observed differences in neural activity are a cause of dyslexia or instead a consequence of the atypical (severely reduced) reading experience that accompanies having dyslexia (Goswami, 2015). Despite being matched for reading experience, and being able to report correctly the same number of words in the sentences as the RL controls, the children with dyslexia showed significantly poorer encoding of speech envelope information in the delta band than the younger RL children, suggestive of a fundamental representational deficit. The accuracy of children's low-frequency envelope encoding was also significantly related to individual differences in phonological awareness (lexical stress perception), consistent with the predictions of temporal sampling theory.

It is important to note that depending on when in the developmental trajectory group differences are measured, and which orthography participants are learning to read, a differential processing balance between hemispheres may characterise individuals with dyslexia at a range of temporal rates, with impairments at some rates and compensation at other rates dependent on cross-language differences in both orthographies and phonologies (e.g., Lehongre et al., 2011, Hämäläinen et al., 2012, Lizarazu et al., 2015). Cross-language tests of temporal sampling theory using identical tasks and comparing both children and adults are needed to throw further light on these issues. Meanwhile, there is a large functional magnetic resonance imaging (fMRI) and positron emission tomography (PET) literature attesting to atypical activation of left hemisphere language networks by participants with dyslexia (Richlan et al., 2013, for a recent summary). Atypical activation of the left-lateralised ‘reading network’ is found in both linguistic and phonological tasks (e.g., reduced activity in left temporo-parietal areas when making phonological judgements, Rumsey et al., 1997, Shaywitz et al., 2002, Brunswick et al., 1999). As previously, it is notable that the majority of relevant studies in the literature are with adults, leaving open the question of whether these left hemisphere differences arise as a consequence of the reduced reading experience that accompanies being dyslexic (Goswami, 2015) or are causal to the disorder. In the only longitudinal neuroanatomical study of developmental dyslexia to date that began *pre-reading*, abnormalities in the left-lateralised reading network were only observed in Norwegian children at-risk for dyslexia *after the children had learned how to read* (Clark et al., 2014). The neuroanatomical precursors to dyslexia were restricted to the primary sensory cortices (Clark et al., 2014). The most consistent group difference in anatomical structure over the course of this longitudinal study was found for Heschl's gyrus, a structure in auditory cortex that is interested in slow temporal modulations (Overath et al., 2015).

Overall, therefore, both neuroanatomical data and the functional neural data presented here are consistent with the neural temporal sampling framework for developmental dyslexia (Goswami, 2011, Goswami, 2015). Accordingly, atypical childhood phase entrainment in the delta band (and possibly the theta band, which was not tested here) to the temporal modulations in speech may be related to the phonological impairments that characterise this disorder of learning, across languages. If neuronal sampling of the speech signal is atypical, then segmentation of the speech stream into meaningful phonological units would also be atypical, from early in development (Goswami and Leong, 2013). For example, stress patterns, stressed syllables and prosodic structure would be identified less effectively. Consequently, individuals with dyslexia may rely more on other speech features that are less dependent on slower modulations in the speech envelope to develop phonological representations for speech recognition and production, at least prior to learning to read. Given that perception of the amplitude envelope is known to be impaired in children with developmental dyslexia across languages (see Goswami, 2015), other acoustic elements such as rapid spectral changes might receive extra weighting in the speech perception of individuals with dyslexia and consequently in the phonological representation (Goswami et al., 2011, for relevant data). Over developmental time, and as orthographic information is integrated into the phonological lexicon via learning to read, these differential weightings could result in a different balance of processing in developmental dyslexia between the two hemispheres in tasks requiring sensitivity to different temporal modulation rates, with patterns of weightings possibly dependent on orthography. In contrast, typically-developing children would develop phonological lexical representations that are optimally organised to support the efficient acquisition of an orthographic system. As they learn a particular orthographic system, typically-developing children may then develop a different balance of hemispheric processing of different temporal modulation rates to children with dyslexia.

## Figures and Tables

**Table 1:** Participant details.

	Dyslexic	Chronological age controls	*F*(1,28)
Chronological age (months) (s.d.)	154.8 (13.9)	154.5 (8.9)	0.1
Reading standard scorea,^b^ (s.d.)	87.5 (12.1)	105.6 (8.7)	22.2⁎⁎⁎
Reading age in months^b^ (s.d.)	128.3 (21.0)	162.6 (20.6)	20.4⁎⁎⁎
Vocabulary standard score^a^ (s.d.)	106.2 (14.6)	109.3 (10.0)	0.5
WISC short-form IQ standard scorea,^c^ (s.d.)	108.3 (14.1)	106.0 (9.6)	0.3
WISC Nonverbal IQ standard score^d^	15.5 (3.6)	13.5 (3.6)	2.4
Mathematics standard score^a^	99.5 (17.3)	103.3 (14.5)	0.4
Phoneme Deletion out of 15^b^ (s.d.)	7.9 (3.0)	11.5 (2.3)	13.5⁎⁎
Rapid Automatized Naming in seconds (s.d.)	44.1 (10.5)	39.2 (5.7)	2.5
Phonological short-term memory^b^ (s.d.)	35.0 (10.8)	50.3 (13.4)	11.9⁎⁎
1 Rise threshold AXB format (ms)	102.9 (79.6)	54.8 (51.8)	3.9+
1 Rise threshold 2IFC format (ms)^b^	93.4 (82.5)	35.8 (10.2)	7.3⁎
Rise Duration Rove threshold (ms)^b^	117.5 (81.0)	53.3 (36.5)	7.7⁎⁎
Barkley Inattention scale^d^	10.7 (6.0)	9.0 (5.8)	0.6
Barkley Hyperactivity scale^d^	6.1 (5.1)	6.0 (4.8)	0

Note. WISC = Wechsler Scale of Intelligence for Children.

+ *p* < 0.10.

⁎ *p* < 0.05.

⁎⁎ *p* < 0.01.

⁎⁎⁎ *p* < 0.001.

a Standard Score = 100, s.d. = 15.

b DYS worse than CA.

c Measured at beginning of longitudinal study.

d Standard score = 10, s.d. = 3.

**Table 2:** Partial correlations between the hemodynamic response for 2 Hz and 40 Hz stimulation and the behavioural measures, controlling for age and IQ.

	1 Rise AXB	Rise Rove	1 Rise 2IFC	BPVS SS	BAS Read SS	Reading Age in months	BAS Maths SS	Phon Deletion	PSTM	RAN	Inatt	Hyper
2 Hz	0.65⁎⁎⁎	0.78⁎⁎⁎	0.57⁎⁎	− 0.53⁎⁎	− 0.42⁎	− .37^a^	− 0.13	− 0.23	− 0.15	0.08	0.19	0.09
40 Hz	0.22	0.32	0.28	− 0.03	− 0.02	0.28	− 0.06	0.04	0.28	0.07	0.33	0.23

⁎ *p* < 0.05.

⁎⁎ *p* < 0.01.

⁎⁎⁎ *p* < 0.001.

a *p* = 0.052.
